# Immune Responses to a Recombinant Glycoprotein E Herpes Zoster Vaccine in Adults Aged 50 Years or Older

**DOI:** 10.1093/infdis/jiy095

**Published:** 2018-02-26

**Authors:** Anthony L Cunningham, Thomas C Heineman, Himal Lal, Olivier Godeaux, Roman Chlibek, Shinn-Jang Hwang, Janet E McElhaney, Timo Vesikari, Charles Andrews, Won Suk Choi, Meral Esen, Hideyuki Ikematsu, Martina Kovac Choma, Karlis Pauksens, Stéphanie Ravault, Bruno Salaun, Tino F Schwarz, Jan Smetana, Carline Vanden Abeele, Peter Van den Steen, Ilse Vastiau, Lily Yin Weckx, Myron J Levin, ZOE-50/70 Study Group

**Affiliations:** 1 The Westmead Institute for Medical Research, University of Sydney, Australia; 2 GSK, King of Prussia, Pennsylvania; 3 GSK, Wavre, Belgium; 4 Faculty of Military Health Sciences, University of Defense, Hradec Kralove, Czech Republic; 5 Department of Family Medicine, Taipei Veterans General Hospital, and National Yang Ming University School of Medicine, Taiwan; 6 Health Sciences North Research Institute, Sudbury, Ontario, Canada; 7 Vaccine Research Center, University of Tampere, Finland; 8 Diagnostics Research Group, San Antonio, Texas; 9 Division of Infectious Disease, Department of Internal Medicine, Korea University College of Medicine, Seoul; 10 Institute of Tropical Medicine, University Clinic of Tuebingen, Germany; 11 Japan Physicians Association, Kanda, Chiyoda-ku, Tokyo; 12 GSK, Rockville, Maryland; 13 Department of Infectious Diseases, Uppsala University Hospital, Sweden; 14 GSK, Rixensart, Belgium; 15 Central Laboratory and Vaccination Centre, Klinikum Würzburg Mitte, Standort Juliusspital, Germany; 16 Federal University of Sao Paulo, Brazil; 17 Department of Pediatrics, University of Colorado Anschutz Medical Campus, Aurora; 17a Department of Medicine, University of Colorado Anschutz Medical Campus, Aurora

**Keywords:** varicella-zoster virus, herpes zoster vaccine, gE subunit vaccine, adjuvant system, immunogenicity

## Abstract

**Background:**

The herpes zoster subunit vaccine (HZ/su), consisting of varicella-zoster virus glycoprotein E (gE) and AS01_B_ Adjuvant System, was highly efficacious in preventing herpes zoster in the ZOE-50 and ZOE-70 trials. We present immunogenicity results from those trials.

**Methods:**

Participants (ZOE-50: ≥50; ZOE-70: ≥70 years of age) received 2 doses of HZ/su or placebo, 2 months apart. Serum anti-gE antibodies and CD4 T cells expressing ≥2 of 4 activation markers assessed (CD4^2+^) after stimulation with gE-peptides were measured in subcohorts for humoral (n = 3293) and cell-mediated (n = 466) immunogenicity.

**Results:**

After vaccination, 97.8% of HZ/su and 2.0% of placebo recipients showed a humoral response. Geometric mean anti-gE antibody concentrations increased 39.1-fold and 8.3-fold over baseline in HZ/su recipients at 1 and 36 months post-dose 2, respectively. A gE-specific CD4^2+^ T-cell response was shown in 93.3% of HZ/su and 0% of placebo recipients. Median CD4^2+^ T-cell frequencies increased 24.6-fold (1 month) and 7.9-fold (36 months) over baseline in HZ/su recipients and remained ≥5.6-fold above baseline in all age groups at 36 months. The proportion of CD4 T cells expressing all 4 activation markers increased over time in all age groups.

**Conclusions:**

Most HZ/su recipients developed robust immune responses persisting for 3 years following vaccination.

**Clinical Trials Registration:**

NCT01165177; NCT01165229.

## Teaser

The herpes zoster subunit vaccine, consisting of varicella-zoster virus glycoprotein E and the AS01_B_ Adjuvant System, stimulated specific antibody and CD4 T-cell responses in >90% of recipients which, in most, persisted for the 36-month duration of the study.

Herpes zoster (HZ) occurs following reactivation of latent varicella-zoster virus (VZV) in sensory and autonomic neurons [1]. Incidence of HZ varies from 6–8 cases/1000 person-years at age 50–59 years of age to >11 cases/1000 person-years at 70 years of age [2–6]. The severity of HZ and its complications also increase with age [2], closely corresponding to the age-related decline in VZV-specific T-cell–mediated immunity (CMI) that is considered important in preventing the reactivation of latent VZV and preventing the propagation of the reactivated virus [7–9]. HZ vaccines are believed to boost VZV-specific memory T cells, preventing their decline below the presently unknown threshold required for protection against HZ [10].

A live attenuated VZV vaccine (Zostavax, Merck Sharpe & Dohme Corp, hereafter referred to as Zoster Vaccine Live [ZVL]), is available to prevent HZ in individuals ≥50 years of age. However, ZVL has some limitations. Clinical trials indicate that vaccine efficacy against HZ is 70% in adults 50–59 years of age, and declines with age from 64% in persons 60–69 years to 18% in those ≥80 years [5, 6]. Moreover, efficacy of ZVL against HZ decreases over time, from 62% in the first year after vaccination to approximately 40% by the fifth year postvaccination [11–13].

A recombinant glycoprotein E (gE) subunit vaccine (HZ/su) was developed to overcome the unmet medical need for a better vaccine. HZ/su consists of the recombinant VZV gE and the AS01_B_ Adjuvant System. gE was selected as the vaccine antigen because it is the most abundant glycoprotein expressed by VZV-infected cells [14] and it induces both neutralizing antibody and CD4 T-cell responses [15–17]. AS01_B_ contains *Quillaja saponaria* Molina, fraction 21 (QS-21; licensed by GSK from Antigenics LLC, a wholly owned subsidiary of Agenus Inc., a Delaware, US corporation) and 3-*O*-desacyl-4′-monophosphoryl lipid A (MPL). AS01_B_ stimulates a local and transient activation of the innate response leading to the recruitment and activation of antigen-presenting dendritic cells [18]. QS-21 is an adjuvant that induces transient local cytokine responses and activation of dendritic cells and macrophages in muscle and draining lymph nodes in animal models [19]. The toll-like receptor type 4 agonist MPL synergizes with QS-21 to enhance the immune response to the coadministered antigen through the production of interferon-gamma (IFN-γ) [20].

Phase I and II trials demonstrated that a single HZ/su dose elicits substantial humoral and CMI responses, which further increase after a second dose. While humoral responses to HZ/su were age-independent, CMI responses declined modestly with age [21–23]. Nonetheless, in adults ≥60 years of age both anti-gE antibody concentrations and gE-specific CD4 T-cell frequencies expressing ≥2 activation markers (CD4^2+^ T cells) remained substantially above prevaccination levels for at least 9 years [24], with statistical models predicting persistence for at least 15 years [25]. Two pivotal phase III efficacy trials of HZ/su in adults ≥50 years of age (ZOE-50) and ≥70 years (ZOE-70) demonstrated age-independent protection against HZ, including 91% protection in vaccinees ≥80 years, and an acceptable safety profile [3, 4]. Overall vaccine efficacy remained high (88%) for the 4-year duration of the ZOE-70 trial [4]. These findings suggest that HZ/su can overcome immunosenescence to provide enduring protection against HZ [26].

This manuscript provides the first comprehensive overview of gE-specific humoral and CMI responses, including polyfunctional CD4^+^ T-cell responses, to HZ/su over a 3-year period, as assessed in subsets of participants from the ZOE-50 and ZOE-70 trials.

## METHODS

### Study Design and Participants

**Figure 1.**
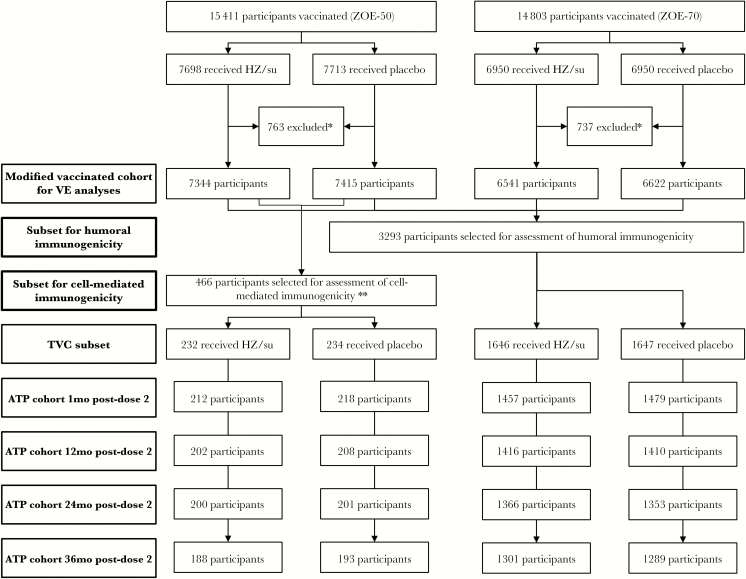
Disposition of study participants in ZOE-50/ZOE-70, zoster efficacy studies in participants ≥50 and ≥70 years of age (trial registration numbers NCT01165177 and NCT01165229). Abbreviations: ATP, according-to-protocol; HZ/su, herpes zoster subunit vaccine; mo, month; TVC, total vaccinated cohort; VE, vaccine efficacy. * details for study exclusion presented elsewhere [3, 4]. ** only participants from the ZOE-50 clinical trial were selected for inclusion in the subset for cell-mediated immunogenicity.

ZOE-50 and ZOE-70 were parallel, phase III, randomized, observer-blind, controlled trials conducted in 18 countries in Europe, North America, Latin America, and Asia/Australia in adults ≥50 years of age (NCT01165177) and ≥70 years of age (NCT01165229), respectively [3, 4]. Participants were randomized 1:1 to receive 2 intramuscular doses 2 months apart of either HZ/su or saline placebo. The total vaccinated cohorts consisted of 15411 participants in ZOE-50 and 13900 in ZOE-70 [3, 4]. A random subset of 3293 ZOE-50 and ZOE-70 participants from all 18 countries was selected for assessment of humoral immune responses. For the assessment of gE-specific CMI responses, 466 ZOE-50 participants from the Czech Republic, Japan, and the United States were randomly selected (Figure 1). Blood was collected for immunological studies on day 0 (prevaccination) and at months 3, 14, 26, and 38 (1, 12, 24, and 36 months post-dose 2, respectively). In both trials, participants in the immunogenicity subsets were stratified by age (50–59, 60–69, 70–79, ≥80) to ensure a balanced age distribution. Detailed descriptions of the trials are presented elsewhere [3, 4].

### Study Vaccine

Each dose of HZ/su (Shingrix, GlaxoSmithKline Biologicals SA) combines 50 μg purified gE with AS01_B_, an adjuvant system containing MPL (50 μg), QS-21 (50 μg) within liposomes [27]. The placebo was saline. As HZ/su and the saline placebo differ in appearance, injections were prepared and administered by study staff not involved in further study assessments.

### Immunogenicity Assessment

Serum anti-gE antibody concentrations were measured using a GSK in-house enzyme-linked immunosorbent assay (ELISA). Details are presented in the Supplementary material.

gE-specific CMI responses were measured using a GSK in-house assay that assessed the frequency of CD4 T cells expressing 2 or more of the following activation markers (hereafter termed CD4^2+^): IFN-γ, interleukin-2 (IL-2), tumor necrosis factor-α (TNF-α), and CD40 ligand (CD40L). Details are presented in the Supplementary material.

### Statistical Analyses

The evaluation of vaccine-induced humoral and CMI responses was an exploratory objective in the ZOE-50 and ZOE-70 trials. The according-to-protocol cohort for immunogenicity at each time point included all participants who received both doses, met all the eligibility criteria, complied with the protocol, and had immunogenicity data available.

Anti-gE antibody geometric mean concentrations (GMCs) and their 95% confidence intervals (CIs) were determined for all vaccine and placebo recipients, and for each age group, in the subsets for humoral and cell-mediated immunogenicity. The humoral response threshold was defined as a ≥4-fold increase in the anti-gE antibody concentration as compared to the prevaccination concentration (for initially seropositive participants) or as compared to the anti-gE antibody cut-off value for seropositivity (97 milli-International Units (mIU)/mL, for initially seronegative participants).

The frequency of gE-specific CD4^2+^ T cells was calculated as the difference between the frequency of CD4^2+^ T cells stimulated in vitro with gE peptides and those stimulated with culture medium alone. The CMI-response threshold was defined as a ≥2-fold increase in the frequency of CD4^2+^ T cells, as compared to prevaccination frequencies (for participants with prevaccination CD4^2+^ T-cell frequencies above the cut-off of 320 positive cells per 10^6^ CD4 T cells counted) or a ≥2-fold increase above the cut-off (for participants with prevaccination frequencies below the cut-off).

Exact 95% CIs were computed at each time point for the percentage of humoral and CMI responders. Medians with interquartile ranges were calculated for CD4^2+^ T-cell frequencies. The 95% CI for GMCs was computed by anti-log transformation of the 95% CI for the mean of log-transformed concentrations (which were calculated assuming that log-transformed values were normally distributed with unknown variance).

Post-hoc analysis on the polyfunctionality of gE-specific CD4^+^ T cells was accomplished by summarizing the median frequencies of CD4^+^ T cells expressing only 1 or any combination of 2, 3, or 4 markers using descriptive statistics. Spearman correlation coefficients were calculated to evaluate the correlation between anti-gE concentrations and gE-specific CD4^2+^ T-cell frequencies.

## RESULTS

### Study Population

Characteristics of the overall study populations of ZOE-50 and ZOE-70 have been published previously and are provided in Supplementary Table 1. HZ/su and placebo recipients in both the humoral and CMI subcohorts did not differ in age, gender, and geographic ancestry (Table 1), but differed from the overall study population in geographic ancestry due to sample selection.

**Table 1.** Demographic Characteristics of Study Participants (According-to-Protocol Cohorts for Immunogenicity)

	Cohort for Humoral Immunogenicity		Cohort for Cell-Mediated Immunogenicity	
	HZ/su (N = 1457)	Placebo (N = 1479)	HZ/su (N = 212)	Placebo (N = 218)
Age (years)				
Mean age at vaccine dose 1, years ± SD	67.5 ± 9.5	67.8 ± 9.5	64.1 ± 9.0	64.5 ± 8.9
50–59, n (%)	356 (24.4)	355 (24.0)	74 (34.9)	73 (33.5)
60–69, n (%)	359 (24.6)	356 (24.1)	68 (32.1)	72 (33.0)
≥70, n (%)			70 (33.0)	73 (33.5)
70–79, n (%)	597 (41.0)	608 (41.1)		
≥80, n (%)	145 (10.0)	160 (10.8)		
**Sex, n (%**)				
Female	852 (58.5)	864 (58.4)	108 (50.9)	119 (54.6)
Male	605 (41.5)	615 (41.6)	104 (49.1)	99 (45.4)
**Geographic ancestry, n (%**)				
White–Caucasian/European	1010 (69.3)	1032 (69.8)	122 (57.5)	128 (58.7)
Asian–East Asian	250 (17.2)	245 (16.6)	1 (0.5)	0 (0.0)
Asian–Japanese Heritage	126 (8.6)	128 (8.7)	72 (34.0)	75 (34.4)
African/African American	31 (2.1)	25 (1.7)	17 (8.0)	14 (6.4)
Other	40 (2.8)	49 (3.3)	0 (0.0)	1 (0.5)

Abbreviations: HZ/su, herpes zoster subunit vaccine; N, number of participants in the group; n (%), number and percentage of participants in a given category; SD, standard deviation.

### Humoral Immunogenicity

#### Response Rate

**Figure 2.**
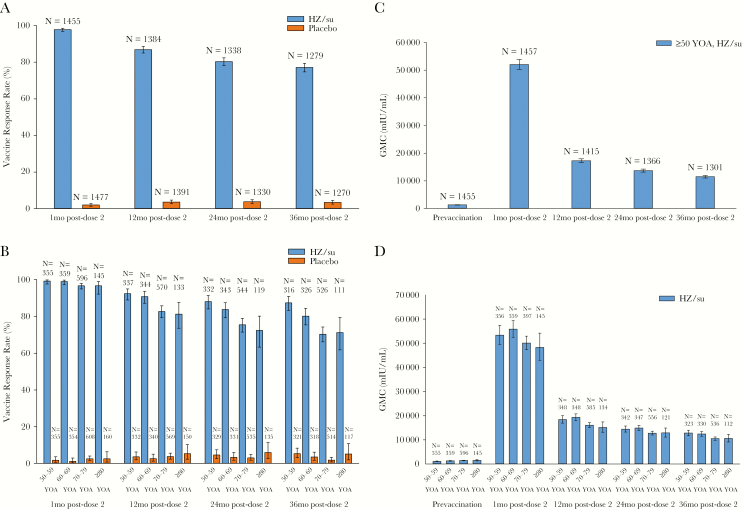
Herpes zoster subunit vaccine (HZ/su)-induced antiglycoprotein E antibody responses (according-to-protocol cohort for humoral immunogenicity): percentage of responders overall (*A*), the percentage of responders by age (*B*), geometric mean concentration (GMCs) overall (*C*), GMCs by age (*D*). Abbreviations: mo, month; N, number of participants with available results; YOA, years of age. Error bars depict 95% confidence intervals.

Prior to vaccination, >99% of participants were seropositive for anti-gE antibodies, and anti-gE antibody GMCs were comparable in the vaccine and placebo groups (Supplementary Table 2). In placebo recipients, there was no significant change in anti-gE antibody GMCs at any time point after vaccination. HZ/su recipients who were seronegative prior to vaccination responded to the vaccine, and 1 month after the second vaccination 97.8% of HZ/su recipients met the criterion for humoral response, compared to 2.0% of placebo recipients. A mean peak response was observed at 1-month post-dose 2. The proportion of HZ/su recipients with a humoral response above the response threshold decreased over time, such that 77.1% of HZ/su recipients remained above the humoral response threshold at 36 months post-dose 2 (Figure 2A). The HZ/su response rate at 1 month post-dose 2 was comparable in HZ/su recipients across all age groups; at 12, 24, and 36 months following vaccination, although the response rate was slightly lower in the older age groups (Figure 2B).

#### GMCs and Fold Increases

Overall, in HZ/su recipients ≥50 years of age, anti-gE antibody GMCs were increased 39.1-fold and 8.3-fold over baseline, at 1 month and 36 months post-dose 2, respectively (Figure 2C, Supplementary Figure 1). Minimal differences in anti-gE antibody GMCs were apparent between age groups at any time point after vaccination (Figure 2D).

### Cell-Mediated Immunogenicity

#### Response Rate

**Figure 3.**
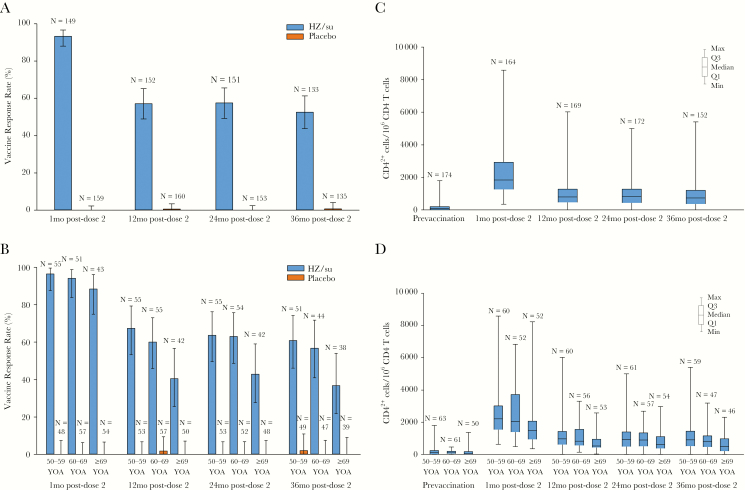
Herpes zoster subunit vaccine (HZ/su)-induced glycoprotein E-specific cell-mediated immunity (according-to-protocol cohort for cell-mediated immunogenicity): percentage of responders overall (*A*), percentage of responder by age (*B*), CD4^2+^ frequencies overall (*C*), CD4^2+^ frequencies by age (*D*). Only HZ/su is shown is panels *C* and *D*. Abbreviations: mo, month; N, number of participants with available results; YOA, years of age. Error bars depict 95% confidence intervals (*A* and *B*) or minimum and maximum values (*C* and *D*).

Prior to vaccination, median gE-specific CD4^2+^ T-cell frequencies were comparable between HZ/su and placebo recipients (Supplementary Table 2). In placebo recipients, there was no significant change in gE-specific CD4^2+^ T-cell frequencies after vaccination at any time point. At 1 month post-dose 2, the CMI-response rate was 93.3% in HZ/su recipients. The proportion of HZ/su recipients above the CMI-response threshold decreased to 57.2% at 12 months post-dose 2 and then remained stable through month 36 (Figure 3A). At 12, 24, and 36 months following vaccination, a slightly lower proportion of HZ/su recipients ≥70 years of age remained above the CMI-response threshold compared to HZ/su recipients <70 years (Figure 3B).

#### CD4^2+^ /CD8 Frequencies and Fold Increases

Overall, in HZ/su recipients ≥50 years of age, the median frequency of gE-specific CD4^2+^ T cells increased 24.6-fold over baseline at 1 month post-dose 2. Median CD4^2+^ T-cell fold increases declined by 12 months post-dose 2, but remained stable thereafter, and at 36 months post-dose 2 were 7.9-fold over baseline (Figure 3C, Supplementary Figure 1). Fold increases in CD4^2+^ T-cell frequencies were 23.0, 24.6, and 33.2-fold over baseline at 1 month post-dose 2 in age groups 50–59, 60–69, and ≥70, respectively. At all time points, median CD4^2+^ T-cell frequencies tended to be lower in HZ/su recipients ≥70 years than in those <70 years (Figure 3D). No significant differences were observed by region.

Scarce gE-specific CD8 T-cell responses were detected in some participants, but these were not increased upon vaccination with HZ/su (data not shown).

#### CD4 T-Cell Polyfunctionality

**Figure 4.**
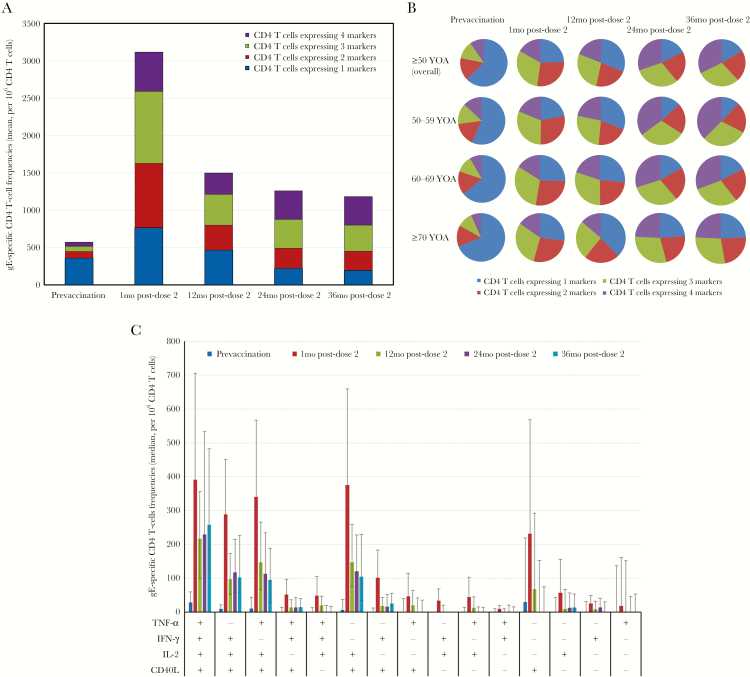
Frequency of CD4^+^ T cells expressing any combination of immune markers (according-to-protocol cohort for cell-mediated immunogenicity): polyfunctional CD4^+^ T-cell frequencies overall (*A*), polyfunctionality proportions by age group (*B*), and activation marker combinations overall (*C*). Abbreviations: mo, month; YOA, years of age. Immune markers: IFN-γ, interferon-γ; IL-2, interleukin-2; TNF-α, tumor necrosis factor-α; CD40L, cluster of differentiation 40 ligand. *B* shows mean percentages. Error bars in *C* depict interquartile ranges.

The mean frequencies of CD4 T cells expressing 2, 3, or 4 activation markers increased considerably over baseline in HZ/su recipients by 1 month post-dose 2. While the frequencies of polyfunctional CD4^+^ T cells had declined by month 12, they remained substantially higher than baseline levels thereafter (Figure 4A). However, this decline was more marked in those expressing only 1 marker, such that during the second and third year post-dose 2, the proportion of CD4 T cells expressing 3 or 4 activation markers (CD4^3+^) increased, both in HZ/su recipients overall and in each age group (Figure 4B). The proportion of T cells expressing only 1 activation marker showed a slight increase from 1 to 12 months after vaccination, but then decreased at 24 and 36 months post-dose 2. Although the proportion of polyfunctional responses was comparable in all age groups, the proportion of CD4^+^ T cells expressing only 1 marker remained slightly higher from 12 months postvaccination onwards in HZ/su recipients ≥70 years of age compared to HZ/su recipients ≤70 years (Figure 4B).

At all time points, CD40L was the most commonly expressed marker, either alone or together with IL-2. IFN-γ and TNF-α were usually expressed in combination with CD40L and/or IL-2. IFN-γ^+^–IL-2^−^ cells appeared at 1 month post-dose 2 and then generally declined to a plateau from month 12 onwards. IFN-γ^−^–IL-2^+^ cells also appeared early but in greater concentrations. Their decline was also proportional to the overall CD4 T-cell population. The same pattern, at a much lower magnitude, was observed with IFN-γ^+^–IL-2^+^ cells (Figure 4C).

### Correlation Between Humoral and Cell-Mediated Immunogenicity

Exploratory analyses in HZ/su recipients at 1 month through 24 months post-dose 2 showed a moderate positive correlation between the humoral and CMI responses, which at 36 months post-dose 2 was weaker but remained statistically significant (Table 2).

**Table 2.** Correlations Between Humoral and Cell-Mediated Immune Responses (According-to-Protocol Cohort for Cell-Mediated Immunogenicity)

Time point	N	Spearman Correlation Coefficient	*P*- value
1 mo post-dose 2	164	0.433	<.0001
12 mo post-dose 2	169	0.3866	<.0001
24 mo post-dose 2	172	0.3287	<.0001
36 mo post-dose 2	152	0.2716	.0007

Correlations were calculated between antiglycoprotein E concentrations and gE-specific CD4^2+^ T-cell frequencies.

Abbreviations: N, number of participants with available results; mo, months.

## DISCUSSION

The 3-year kinetics of gE-specific antibody and CMI responses after immunization with HZ/su were determined in specific subsets of participants ≥50 years of age from the 2 pivotal phase III efficacy trials.

Over 99% of participants had detectable gE-specific antibodies at baseline, and concentrations were similar across age groups. A peak in anti-gE antibody concentrations was observed 1 month following dose 2 and then declined, consistent with previous observations [28]. Antibody concentrations remained above the humoral response threshold in >75% of vaccinees at 36 months following dose 2. Humoral responses were elevated in all age groups, with anti-gE GMCs only slightly smaller in participants >70 years of age throughout the 36 months of observation. This is consistent with findings from earlier studies in which VZV-specific humoral immunity was observed to be largely age independent [7].

While a high, persistent circulating antibody response to HZ/su is induced, gE-specific CMI is believed to be the main mechanistic driver of protection against HZ [8]. In line with results from phase II clinical trials [21–23], HZ/su induced a gE-specific CMI response in >90% of recipients. Peak CD4^2+^ T-cell frequencies were observed at 1 month following dose 2, then declined substantially by 12 months after dose 2, and remained stable for the remainder of the study. Postvaccination median fold increases in CD4^2+^ T-cell frequencies were higher in HZ/su recipients ≥70 compared to those <70 years of age, although median frequencies in recipients ≥70 were lower at all time points. The higher fold increase in those ≥70 years resulted from a lower median baseline value. Thirty-six months following dose 2, CD4^2+^ T-cell responses were still above the CMI response threshold in half of the HZ/su vaccinees, consistent with results of the long-term follow-up of a phase II study that demonstrated persistence of these responses for at least 9 years [24].

The kinetics of gE-specific CMI responses are comparable to VZV-specific responses to ZVL over a 3-year observation period despite differences in the assays used [7]. However, HZ/su induced much greater fold increases in humoral and cellular responses than ZVL. The higher magnitude of the immune response to HZ/su could contribute to the difference in efficacy of the vaccines [3–6]. The ability of HZ/su to elicit this substantial immune response in the older age groups is likely due to the capacity of the AS01_B_ Adjuvant System to enhance gE-antigen presentation by increasing the number of activated antigen-presenting cells [18, 29]. In addition, AS01 promotes T-cell responses through a synergistic effect between MPL and QS-21, involving the stimulation of macrophages in the draining lymph node and early IFN-γ production, which in turn mediates the effects on dendritic cells [20].

In contrast to ZVL, which induces a broad response against multiple antigens, the immune response to HZ/su is directed against a single immunodominant antigen, indicating that a strong, narrowly focused immune response can be highly protective, even against a complex viral pathogen that possesses multiple immune evasion pathways [30]. On the other hand, the broad immune response elicited by ZVL did not provide the same level of protection, possibly because the response is of insufficient magnitude and/or because many of the vaccine antigens do not elicit protective immune responses.

In addition to the robust increase in CD4^2+^ T-cell frequencies in all age groups, the proportion of polyfunctional CD4^+^ T cells expressing 2 or more activation markers compared to activated CD4^+^ T cells expressing a single activation marker was considerably increased 1 month following dose 2 in HZ/su recipients. In all age groups, the proportion of CD4^+^ T cells expressing only 1 marker decreased and the proportion of polyfunctional CD4^+^ T cells greatly increased from 1 to 24 months after vaccination, and continued to increase at 24 and 36 months following dose 2, with more than half of all gE-specific CD4 T cells expressing at least 3 activation markers. Although the proportion of CD4^3+^ T cells appeared to be greater in HZ/su recipients <70 years, the proportion of CD4^3+^ T cells was over 50% in all age groups at 2 and 3 years after 2 doses of HZ/su. In human trials, CD4^+^ T-cell polyfunctionality correlated with protection induced by many vaccines, including human immunodeficiency virus (HIV), tuberculosis (BCG vaccine), malaria, and melanoma [31–33, S1]. The correlation of CD4^+^ T-cell polyfunctionality with protection has similarly been observed in animal models of vaccines for simian immunodeficiency virus and herpes simplex virus [31–35]. In vaccine trials against metastatic melanoma, T-cell polyfunctionality correlated with long-term survival [36]. The predominant expression of CD40L alone and in 2-, 3-, or 4-marker combinations is consistent with previous findings [23, 37], and mirrors responses to the hepatitis B surface antigen adjuvanted with AS01_B_ that showed CD40L to be the dominant activation marker shortly after vaccination [38]. AS01_B_ in humans and animal models has been shown to induce IFN-γ from CD4 T and NK cells [20]. This cytokine is an essential antiviral component of the immune response to VZV and closely related herpes simplex infections [39–41].

In primary immunization, the initial phase of naive effector CD4 T-cell expansion is followed by marked contraction via apoptosis and concurrent expansion and persistence of memory T cells [42]. This has not been well defined in secondary immunization, relevant to zoster immunization. For example, with tetanus boosters, the expansion phase consists predominantly of effector memory T cells [43] but after ZVL vaccination it is unclear whether naive or effector memory T cells predominate in the expansion phase [41, 44, 45]. In our study, the declining kinetics of CD4^2+^ T cells from 1 month after immunization appears to correspond to the contraction phase although migration into tissues may also contribute.

In immunologic characterization of ZVL, the magnitude and kinetics of effector memory responses at baseline, and after ZVL, were features that distinguished younger and older participants [46]. A recent immunologic comparison of HZ/su and ZVL demonstrated that, 1 year after vaccination, VZV-specific central memory and effector memory CD4^+^ T cells were increased significantly more and persisted longer in HZ/su recipients [45]. These are the cells that might secrete IL-2 and antiviral IFN-γ at the site of reactivating VZV infection.

In our study, the expression of IFN-γ alone, or in combination with IL-2, which has previously been used to differentiate effector and memory T-cell responses, respectively [47], suggests the predominant persistence of central memory (IL-2^+^) and effector memory (IL-2^+^–IFN-γ^+^) CD4^+^ T cells in HZ/su recipients for at least 36 months after immunization. Confirmation of this hypothesis and characterization of the expansion phase will require in-depth phenotyping studies.

Regarding the relatively age-independent CMI response to HZ/su, previous studies in both mice and humans have shown an age-related decline in IL-2 production by CD4 T cells. The addition of IL-2 and proinflammatory cytokines, including IL-6, restores memory CD4 T-cell responses to that observed in young animals (reviewed in [48]), as well as memory CD8 T-cell responses in a CD4-dependent manner [49]. The persistence of strong gE-specific central memory CD4 T-cell responses and subsequent IL-2 production seen in our trials could therefore explain the protection against HZ associated with HZ/su vaccination.

The observed kinetics of the humoral and cellular immune responses to HZ/su were comparable, and were characterized by peak levels at 1 month after dose 2 and a rapid decrease followed by a slow decline or a plateau from 1 year postvaccination onward to maintain a long-term response level. A similar pattern was observed in trials of the herpes simplex virus type 2 (HSV-2) glycoprotein D (gD-2) vaccine [50]. This vaccine is adjuvanted with AS04, an adjuvant system that, like AS01, contains the toll-like receptor 4 agonist MPL, which is known to stimulate B-cell help through follicular helper T cells in draining lymph nodes. We found a moderate correlation between humoral and CD4^2+^ T-cell responses during the first years after HZ/su vaccination, but the strength of the correlation decreased over the whole postvaccination period, the converse of the increasing proportions of polyfunctional CD4^+^ T cells. This could suggest a multiphasic response, possibly with early T-cell help for a humoral immune response followed by later predominance of a persistent specific polyfunctional Th1 CD4 memory T-cell response. These correlations, and their potential implications for vaccine efficacy, should be explored further with a larger number of participants.

In conclusion, 2 doses of HZ/su induced robust humoral and cellular immune responses in all age groups (especially people ≥70 years) that remained substantially above baseline 3 years after vaccination. The ability of this vaccine to induce such persistent antibody and polyfunctional CD4 T-cell responses in older adults are likely important mechanisms by which HZ/su drives the high efficacy against HZ. HZ/su demonstrates that the use of AS01_B_ in the vaccine can overcome immunosenescence. We therefore consider HZ/su to be an important step in the design of future vaccines in this age group. Further studies will be needed to determine the precise underlying mechanisms.

## Supplementary Data

Supplementary materials are available at *The Journal of Infectious Diseases* online. Consisting of data provided by the authors to benefit the reader, the posted materials are not copyedited and are the sole responsibility of the authors, so questions or comments should be addressed to the corresponding author.

Supplementary Figure 1

Supplementary Materials

Supplementary Table 1

Supplementary Table 2

## Notes

***Authors’ contribution.*** Detailed authors’ contribution is provided in the supplementary materials.

***Acknowledgments.*** The authors would like to thank all study participants, the clinical investigators and study nurses involved in the ZOE-50/70 trials, as well as the Clinical Immunology Platform (GSK) for the data analyses and interpretation. Medical writing services were provided by Jarno Jansen, Mihai Suteu, and Alpár Pöllnitz (XPE Pharma & Science on behalf of GSK). Editorial assistance and publication coordination were provided by Quentin Deraedt and Myriam Wilbaux (XPE Pharma & Science on behalf of GSK).

***Financial support.*** This work was supported by GlaxoSmithKline Biologicals SA. GlaxoSmithKline Biologicals SA was involved in all stages of the conduct and analysis of the studies. GlaxoSmithKline Biologicals SA covered the costs associated with the development and the publishing of the present manuscript.

***Trademarks.*** Zostavax is a registered trademark of Merck Sharpe & Dohme Corp. Shingrix is a registered trademark of GlaxoSmithKline Biologicals SA.

***Potential conflicts of interest.*** M. K. C., S. R., B. S., C. V. A., P. V. dS., and I. V. are employees, and T. C. H., H. L., and O. G. are former employees, of the GSK group of companies. T. C. H., H. L., O. G., M. K. C., B. S., and P. V. dS. hold shares or stock options from GSK as part of their current or former employee remuneration. A. L. C. received honoraria paid to his institution from GSK, Merck Sharp & Dohme (Merck), and BioCSL/Sequirus outside the submitted work. T. C. H. is a consultant for GSK and is the coinventor of a patent application related to the vaccine used in this study. H. L. is a current employee of Pfizer and receives stock options as part of his employee remuneration. R. C. reports receiving lecture fees from Pfizer outside the submitted work. J. E. M. received honoraria and fees paid to her institution from GSK, Sanofi Pasteur, Merck, and Pfizer, as well as travel support from Sanofi Pasteur, Merck, and Pfizer outside the submitted work. T. V., W. S. C., and M. E. received fees paid to their institutions from GSK to perform the study. T. V. received lecture fees from GSK outside the submitted work. W. S. C. received grant support from Merck and AbbVie Korea outside the submitted work, and lecture fees from GSK, Pfizer, Merck, SK Chemicals, and Green Cross. M. E. received grants from the Federal Ministry of Education and Research, German Research Foundation, and Baxter outside the submitted work. H. I. received grants and personal fees from GSK and grants from Japan Vaccine during the conduct of the study, as well as grants and personal fees from Daiichi-Sankyo and grants from Sanofi Pasteur and personal fees from Shionogi outside the submitted work. T. F. S. received personal fees from GSK, Pfizer, and Sanofi Pasteur outside the submitted work. J. S. received personal fees from GSK and Pfizer outside the submitted work. L. Y. W. received grant from GSK during the conduct of the study. M. J. L. received fees for serving on advisory boards from Merck and GSK, grant support from Merck and GSK, and royalties from a patent related to a zoster vaccine held with Merck. S. J. H., C. A., and K. P. declare no conflict of interest. All authors have submitted the ICMJE Form for Disclosure of Potential Conflicts of Interest. Conflicts that the editors consider relevant to the content of the manuscript have been disclosed.

***Presented in part.*** IDWeek, 26–30 October 2016, New Orleans, LA; International Herpesvirus Workshop, 23–27 July 2016, Madison, WI; World Congress of Geriatrics and Gerontology, 18–20 November 2016, Kaohsiung (Taiwan); congress of the Japan Association of Infectious Diseases, 8 April 2017, Tokyo, (Japan); Advances and Controversies in our Understanding of Herpes Zoster Royal Society of Medicine, 30–31 March 2017, London (UK); and Vaccines and Related Biological Products Advisory Committee Meeting, 13 September 2017, Silver Spring, MD.
