# Supplementary material for: Immune Responses to a Recombinant Glycoprotein E Herpes Zoster Vaccine in Adults Aged 50 Years or Older
Source: J Infect Dis. 2018 Feb 26;217(11):1750–60. doi: 10.1093/infdis/jiy095 (PMC5946839; doi:10.1093/infdis/jiy095)
Supplement: Supplementary Figure 1 [file jiy095_suppl_supplementary_figure_1.docx]

**Figure S1 – Median anti-gE concentration and median gE-specific CD4^2+^ frequency fold increases over pre-vaccination levels (according-to-protocol cohorts for immunogenicity)**


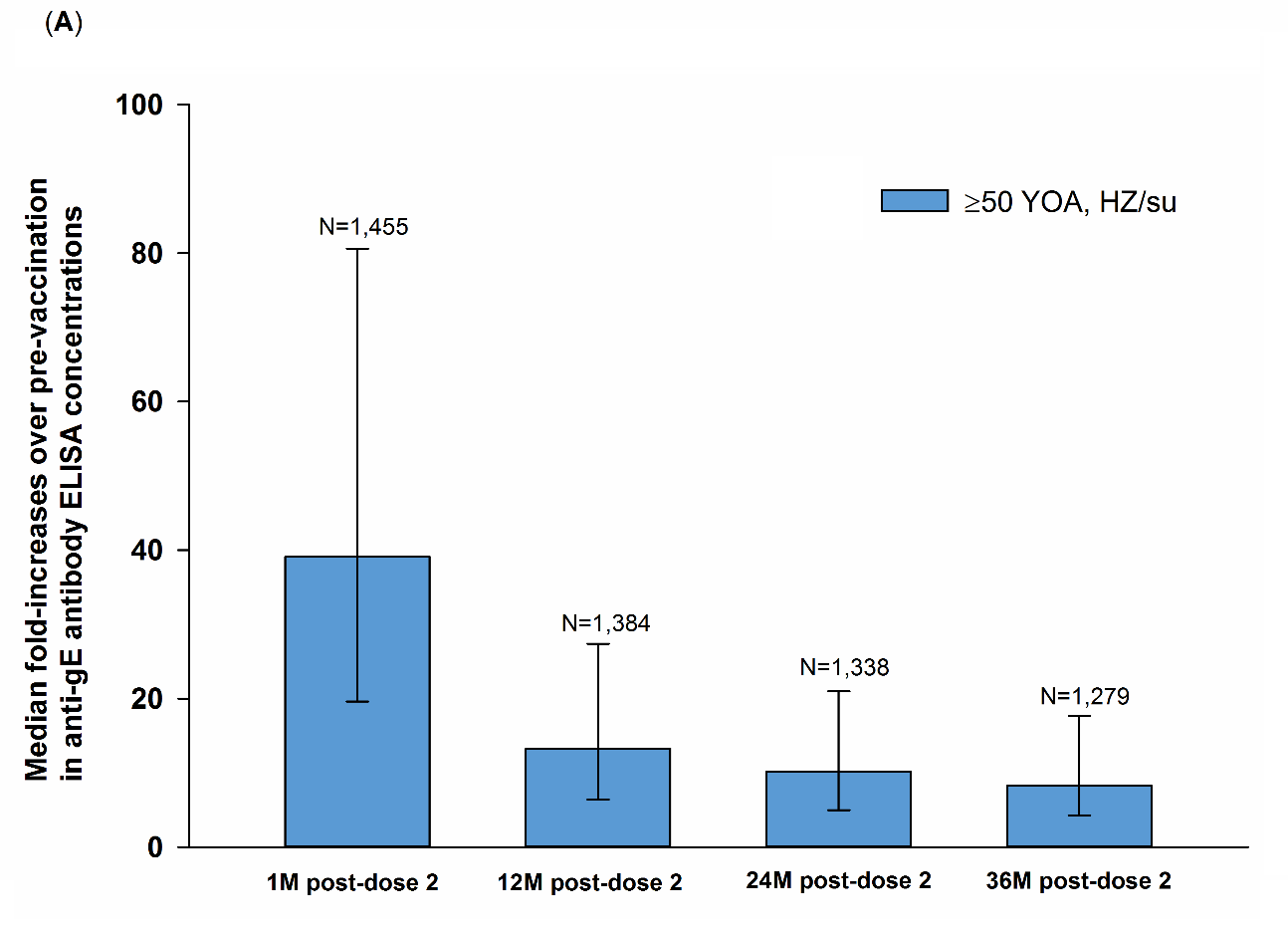


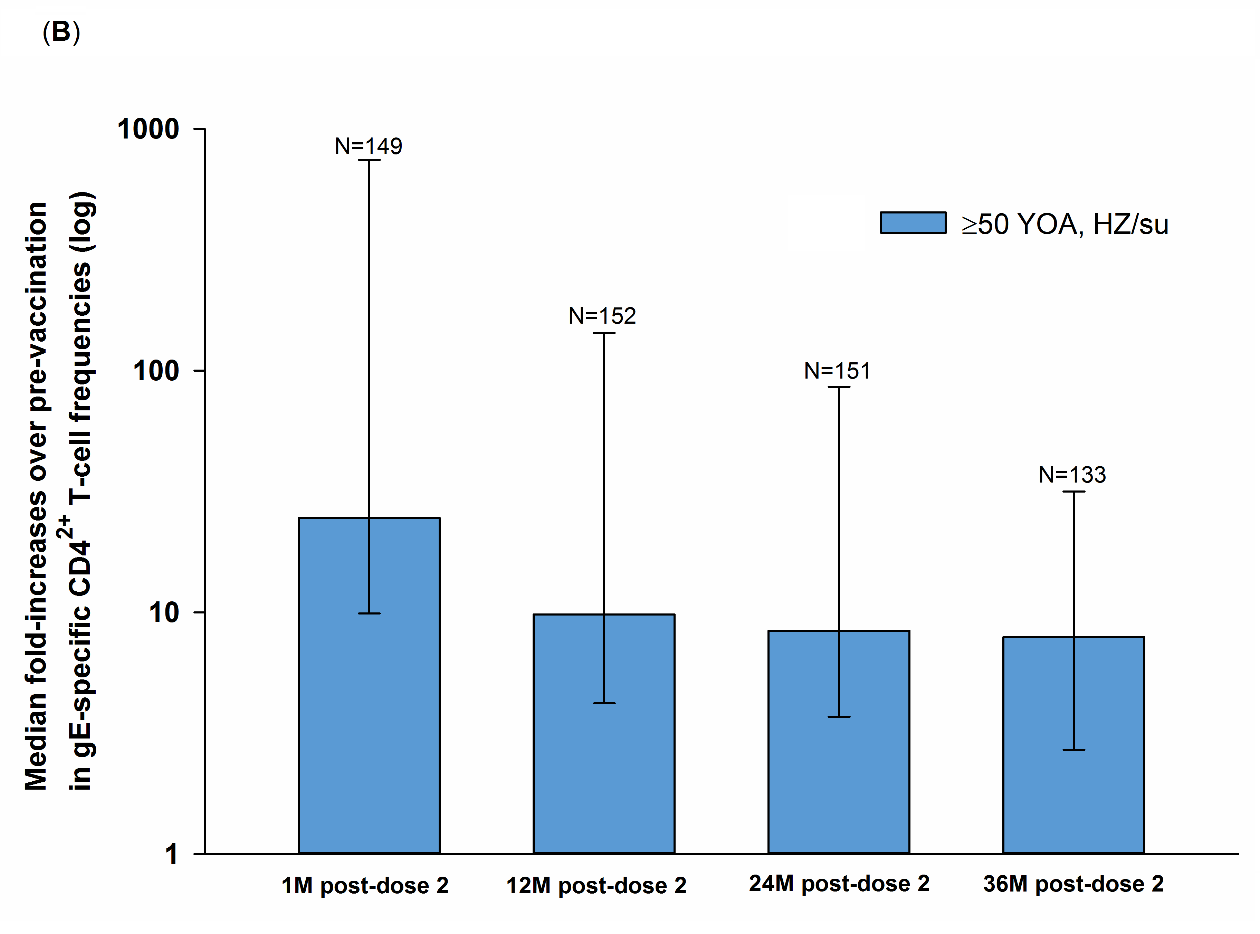


gE, glycoprotein E; ELISA, Enzyme-Linked Immunosorbent Assay; HZ/su, Herpes Zoster subunit vaccine; YOA, years of age; N, number of participants in the group; M, month.

Only HZ/su data are presented. Error bars depict interquartile ranges.
