# Supplementary material for: Immune Responses to a Recombinant Glycoprotein E Herpes Zoster Vaccine in Adults Aged 50 Years or Older
Source: J Infect Dis. 2018 Feb 26;217(11):1750–60. doi: 10.1093/infdis/jiy095 (PMC5946839; doi:10.1093/infdis/jiy095)
Supplement: Supplementary Materials [file jiy095_suppl_supplementary_materials.docx]

**Supplementary Materials**

**Members of ZOE-50/70 study group (by country, in alphabetical order)***

Australia: Eugene Athan, Anthony L. Cunningham, Ferdinandus de Looze, Wilfred Yeo

Brazil: Thiago Junquera Avelino-Silva, Jose Luiz Neto, Lily Yin Weckx

Canada: Wayne Ghesquiere, Iris Gorfinkel, Janet E. McElhaney, Shelly A. McNeil, Azhar Toma

Czech Republic: Roman Chlibek, Jan Smetana

Estonia: Airi Poder

Finland: Tiina Korhonen, Timo Vesikari

Germany: Meral Esen, Tino F Schwarz

Italy: Maria Guiseppina Desole, Tommaso Staniscia

Japan: Hideyuki Ikematsu, Daisuke Watanabe

Korea: Won Suk Choi

Mexico: Abiel Mascarenas de Los Santos, Juan Carlos Tinoco

Spain: Carlos Brotons Cuixart, Covadonga Caso, Silvia Narejos Perez, Maria Luisa Rodriguez de la Pinta

Sweden: Johan Berglund, Karlis Pauksens, Lars Rombo

Taiwan: Shinn-Jang Hwang

United States: Charles Andrews, Myron Levin

GSK (current and former affiliation at the time of the study): Emmanuel Di Paolo, Olivier Godeaux, Thomas C. Heineman, Martina Kovac, Himal Lal, Lidia Oostvogels, Stéphanie Ravault, Bruno Salaun, Carline Vanden Abeele, Peter Van den Steen, Ilse Vastiau, Toufik Zahaf

* Only investigators who agreed to participate in the publication are listed here; therefore, not all countries are represented.

**Authors’ and study group contribution**

*Conceived and designed the study:* Olivier Godeaux, Thomas C. Heineman, Himal Lal, and

Toufik Zahaf.

*Collected the data:* Charles Andrews, Eugene Athan, Thiago Junqueira Avelino-Silva, Johan Berglund, Carlos Brotons Cuixart, Covadonga Caso, Roman Chlibek, Won Suk Choi, Anthony L. Cunningham, Ferdinandus de Looze, Emmanuel Di Paolo, Meral Esen, Wayne Ghesquiere, Iris Gorfinkel, Maria Guiseppina Desole, Shinn-Jang Hwang, Hideyuki Ikematsu, Tiina Korhonen, Abiel Mascarenas de Los Santos, Janet E. McElhaney, Shelly A. McNeil, Silvia Narejos Perez, Jose Luiz Neto, Karlis Pauksens, Airi Poder, Maria Luisa Rodriguez de la Pinta, Lars Rombo, Tino F Schwarz, Jan Smetana, Tommaso Staniscia, Juan Carlos Tinoco, Azhar Toma, Timo Vesikari, Lily Yin Weckx and Wilfred Yeo.

*Analyzed the data:* Emmanuel Di Paolo, Bruno Salaun, Carline Vanden Abeele and Toufik Zahaf.

*Interpreted the data:* Eugene Athan, Roman Chlibek, Anthony L. Cunningham, Ferdinandus de Looze, Emmanuel Di Paolo, Wayne Ghesquiere, Olivier Godeaux, Iris Gorfinkel, Thomas C. Heineman, Shinn-Jang Hwang, Tiina Korhonen, Martina Kovac, Himal Lal, Myron J. Levin, Janet E. McElhaney, Shelly A. McNeil, Lidia Oostvogels, Stéphanie Ravault, Lars Rombo, Bruno Salaun, Jan Smetana, Carline Vanden Abeele, Peter Van den Steen, Ilse Vastiau, Timo Vesikari, Daisuke Watanabe, Lily Yin Weckx, Wilfred Yeo and Toufik Zahaf.

*Wrote the paper:* Roman Chlibek, Anthony L. Cunningham, Thomas C. Heineman, Shinn-Jang Hwang, Himal Lal, Myron J. Levin, Janet E. McElhaney and Timo Vesikari.

All authors reviewed and approved the final submitted version of the paper. All group contributors had the opportunity to review a draft of the paper.

**Material and Methods**

### Recombinant gE

The recombinant gE was produced in CHO cell line. The engineering, protein characterization and protein purification process were performed as previously described [Dendouga et al. 2012].

*Immunogenicity assessment*

Serum anti-glycoprotein E (gE) antibody concentrations were measured using a GSK in-house enzyme-linked immunosorbent assay (ELISA).

Diluted serum samples were added to ELISA 96-well microplates pre-coated with purified recombinant gE antigen. To this, peroxidase-conjugated anti-human immunoglobulin G (IgG) antibodies were added. After incubation of the microtiter wells with a chromogen substrate solution, the enzymatic reaction was stopped and anti-gE antibody concentration were calculated based on a reference standard curve calibrated against the anti-varicella zoster virus (VZV) 90/690 reference (National Institute for Biological Standards and Control). The cut-off for the analysis was 97 milli-International Units (mIU)/mL.

gE-specific CMI responses were measured using a GSK in-house assay.

We assessed the frequency of CD4 T cells expressing the following activation markers: interferon-γ (IFN-γ), interleukin-2 (IL-2), tumor necrosis factor-α (TNF-α), and CD40 ligand (CD40L). Peripheral blood mononuclear cells (PBMCs) were separated from heparinized blood over Ficoll-hypaque, frozen, cryopreserved and stored at -196°C before testing [Weinberg et al. 2009]. Viability of thawed PBMCs, checked prior to testing by intracellular cytokine staining, was required to be >80% [Moris et al. 2011]. PBMCs were stimulated for 2 hours with a pool of 134 15-mer peptides overlapping by 11 (1.25 μg/mL each) spanning the entire gE ectodomain (residues 1 to 546) (Eurogentec), before 18-hour overnight incubation with brefeldin A (1μg/mL) at 37 °C. The PBMC preparation was performed by certified operators, and the certification process includes stimulation with Staphylococcal Enterotoxin B to ensure immunocompetence of separated cells. Cells were stained with a viability dye and for phenotypic surface markers (CD4, CD8), fixed, permeabilized, and stained with antibodies to CD3, CD40L, IFN-γ, TNF- α and IL-2. Cells were then washed and analyzed by flow cytometry, as previously described [Moris et al. 2011]. CMI responses were expressed as the frequency of viable CD4 T cells expressing two or more activation markers (CD4^2+^ T cells) per 10^6^ CD4 T cells. The cut-off for the analysis was 320 positive cells per 10^6^ CD4 T cells counted.

Dendouga N, Fochesato M, Lockman L, Mossman S, Giannini SL. Cell-mediated immune responses to a varicella-zoster virus glycoprotein E vaccine using both a TLR agonist and QS21 in mice. Vaccine 2012; 30:3126-35.

Weinberg A, Song LY, Wilkening C, et al. Optimization and limitations of use of cryopreserved peripheral blood mononuclear cells for functional and phenotypic T-cell characterization. Clin Vaccine Immunol **2009**; 16:1176-86.

Moris P, van der Most R, Leroux-Roels I, et al. H5N1 influenza vaccine formulated with AS03 A induces strong cross-reactive and polyfunctional CD4 T-cell responses. J Clin Immunol **2011**; 31:443-54.

**Supplementary reference**

S1. Thakur A, Pedersen LE, Jungersen G. Immune markers and correlates of protection for vaccine induced immune responses. Vaccine **2012**; 30:4907-20.
