# Supplementary material for: Immune Responses to a Recombinant Glycoprotein E Herpes Zoster Vaccine in Adults Aged 50 Years or Older
Source: J Infect Dis. 2018 Feb 26;217(11):1750–60. doi: 10.1093/infdis/jiy095 (PMC5946839; doi:10.1093/infdis/jiy095)
Supplement: Supplementary Table 1 [file jiy095_suppl_supplementary_table_1.docx]

**Table S1 – Demographic characteristics of the ZOE-50 and ZOE-70 study participants (Total Vaccinated Cohorts)**

|  | | **ZOE-50** | | |  | **ZOE-70** | | |
| --- | --- | --- | --- | --- | --- | --- | --- | --- |
| **Characteristics** | | **HZ/su group (N=7,698)** | **Placebo group (N=7,713)** | **Total (N=15,411)** |  | **HZ/su group (N=6,950)** | **Placebo group (N=6,950)** | **Total (N=13,900)** |
| **Age** | |  |  |  |  |  |  |  |
|  | Mean age at first dose, years ± SD | 62.4 ± 9.0 | 62.3 ± 9.0 | 62.3 ± 9.0 |  | 75.6 ± 4.7 | 75.6 ± 4.7 | 75.6 ± 4.7 |
|  | 50–59 years, n (%) | 3,645 (47.3) | 3,644 (47.2) | 7,289 (47.3) |  |  |  |  |
|  | 60–69 years, n (%) | 2,244 (29.2) | 2,246 (29.1) | 4,490 (29.1) |  |  |  |  |
|  | ≥70 years, n (%) | 1,809 (23.5) | 1,823 (23.7) | 3,632 (23.6) |  |  |  |  |
|  | 70–79 years, n (%) |  |  |  |  | 5,414 (77.9) | 5,420 (78.0) | 10,834 (77.9) |
|  | ≥80 years, n (%) |  |  |  |  | 1,536 (22.1) | 1,530 (22.0) | 3,066 (22.1) |
| **Sex, n (%)** | |  |  |  |  |  |  |  |
|  | Female | 4,711 (61.2) | 4,713 (61.1) | 9,424 (61.2) |  | 3,789 (54.5) | 3,836 (55.2) | 7,625 (54.9) |
|  | Male | 2,987 (38.8) | 3,000 (38.9) | 5,987 (38.8) |  | 3,161 (45.5) | 3,114 (44.8) | 6,275 (45.1) |
| **Race, n (%)*** | |  |  |  |  |  |  |  |
|  | White | 5,532 (71.9) | 5,535 (71.8) | 11,067 (71.8) |  | 5,347 (76.9) | 5,348 (76.9) | 10,695 (76.9) |
|  | Black | 140 (1.8) | 130 (1.7) | 270 (1.8) |  | 79 (1.1) | 67 (1.0) | 146 (1.1) |
|  | Asian | 1,466 (19.0) | 1,470 (19.1) | 2,936 (19.1) |  | 1,216 (17.5) | 1,218 (17.5) | 2,434 (17.5) |
|  | Other | 560 (7.3) | 578 (7.5) | 1,138 (7.4) |  | 308 (4.4) | 317 (4.6) | 625 (4.5) |
| **Region, n (%)** | |  |  |  |  |  |  |  |
|  | Asia or Australia | 1,642 (21.3) | 1,642 (21.3) | 3,284 (21.3) |  | 1,317 (18.9) | 1,319 (19.0) | 2,636 (19.0) |
|  | Europe | 3,941 (51.2) | 3,948 (51.2) | 7,889 (51.2) |  | 3,758 (54.1) | 3,753 (54.0) | 7,511 (54.0) |
|  | Latin America | 772 (10.0) | 779 (10.1) | 1,551 (10.1) |  | 537 (7.7) | 538 (7.7) | 1,075 (7.7) |
|  | North America | 1,343 (17.5) | 1,344 (17.4) | 2,687 (17.4) |  | 1,338 (19.3) | 1,340 (19.3) | 2,678 (19.3) |

HZ/su, Herpes Zoster subunit vaccine; N, number of participants in the group; n (%), number and percentage of participants in a given category; SD, standard deviation.

* race was self-reported
