# Supplementary material for: Immune Responses to a Recombinant Glycoprotein E Herpes Zoster Vaccine in Adults Aged 50 Years or Older
Source: J Infect Dis. 2018 Feb 26;217(11):1750–60. doi: 10.1093/infdis/jiy095 (PMC5946839; doi:10.1093/infdis/jiy095)
Supplement: Supplementary Table 2 [file jiy095_suppl_supplementary_table_2.docx]

**Table S2 – Pre-vaccination immune parameters (according-to-protocol cohorts for immunogenicity)**

|  | | **Anti-gE GMC*** | |  | **Median CD4^2+^ frequency**** | |
| --- | --- | --- | --- | --- | --- | --- |
|  | | **HZ/su** | **Placebo** |  | **HZ/su** | **Placebo** |
|  | | **N=1646** | **N=1647** |  | **N=232** | **N=234** |
| **Age groups** | |  |  |  |  |  |
|  | Overall | 1320.5 (1253.6–1391.0) | 1363.9 (1295.6–1435.8) |  | 89.8 (1.0–202.4) | 81.6 (11.8–215.5) |
|  | 50–59 years | 1097.9 (987.2–1220.9) | 1216.4 (1093.6–1353.2) |  | 110.9 (2.1–241.2) | 66.9 (1.0–179.7) |
|  | 60–69 years | 1301.2 (1179.7–1435.2) | 1310.0 (1184.1–1449.4) |  | 95.8 (30.4–195.9) | 93.2 (21.5–202.6) |
|  | ≥70 years |  |  |  | 49.6 (1.0–172.2) | 98.4 (19.5–249.6) |
|  | 70–79 years | 1440.4 (1325.9–1564.7) | 1442.5 (1331.9–1562.2) |  |  |  |
|  | ≥80 years | 1506.3 (1273.7–1781.3) | 1553.8 (1317.1–1833.1) |  |  |  |

gE, glycoprotein E; GMC, Geometric Mean Concentration; CD4^2+^, gE-specific activated CD4^+^ T cells expressing ≥2 activation markers; HZ/su, Herpes Zoster subunit vaccine; N, number of participants in the group.

* Geometric mean concentrations are presented with 95% confidence intervals

** Median CD4^2+^ frequencies present frequency per 10^6^ cells counted and are presented with interquartile range
